# Self-perceived burden, perceived burdensomeness, and suicidal ideation in patients with chronic pain

**DOI:** 10.1080/24740527.2017.1368009

**Published:** 2017-09-18

**Authors:** Keith G. Wilson, John Kowal, Sara M. Caird, Dyana Castillo, Lachlan A. McWilliams, Adam Heenan

**Affiliations:** a Department of Psychology, The Ottawa Hospital, Ottawa, Ontario, Canada; b Clinical Epidemiology Program, The Ottawa Hospital Research Institute, Ottawa, Ontario, Canada; c Department of Medicine, University of Ottawa, Ottawa, Ontario, Canada; d School of Psychology, University of Ottawa, Ottawa, Ontario, Canada; e Department of Anaesthesiology, University of Ottawa, Ottawa, Ontario, Canada; f Department of Psychology, University of Saskatchewan, Saskatoon, Saskatchewan, Canada; g University of Ottawa Heart Institute, Ottawa, Ontario, Canada

**Keywords:** chronic pain, perceived burdensomeness, self-perceived burden, suicidal ideation

## Abstract

**Background**: Self-perceived burden and perceived burdensomeness are two apparently related constructs that have arisen independently from research in physical and mental health, respectively. Although both are associated with suicidal ideation in individuals with chronic pain, they have yet to be examined concurrently in the same group of patients.

**Aims**: The aim of this study was to investigate the relationship between the two constructs and their differential prediction of suicidal ideation.

**Methods**: Participants were 260 outpatients of an interdisciplinary chronic pain treatment program. Each participant completed the Self-Perceived Burden Scale (SPBS), the Interpersonal Needs Questionnaire Perceived Burdensomeness Scale (INQPBS), the Beck Scale for Suicide Ideation, and the thoughts of self-harm item of the Patient Health Questionnaire-9.

**Results**: The SPBS and the INQPBS were both unifactorial measures with good internal consistency. They correlated significantly with one another (*r* = 0.50, *P* < 0.001), as well as with scores on the two measures of suicidal ideation (*r*s ranging from 0.29 to 0.62, *P*s < 0.001). However, the INQPBS correlated more highly with suicidal ideation than did the SPBS. In regression analyses, the INQPBS predicted unique variance in suicidal ideation after adjusting for the SPBS. Conversely, the SPBS did not contribute uniquely when the INQPBS was entered first.

**Conclusions**: Patients with chronic pain who believe that they have become “a burden to others” are at increased risk for suicidal ideation. The conceptual similarities and differences between the constructs of self-perceived burden and perceived burdensomeness are reviewed to explain why perceived burdensomeness is the stronger predictor of this increased risk.

## Résumé

**Contexte:** La perception de soi comme un fardeau et le fardeau perçu sont deux construits connexes qui ont respectivement émergé de travaux de recherche en santé physique et en santé mentale, de manière indépendante. Bien qu’ils soient tous deux associés à l’idéation suicidaire chez les individus souffrant de douleur chronique, ils n’ont jamais été évalués simultanément au sein du même groupe de patients. **Objectifs:** Examiner le lien entre les deux construits et leur prédiction différentielle de l’idéation suicidaire. **Méthodes:** Le groupe de participants était composé de 260 patients externes d’un programme interdisciplinaire de gestion de la douleur chronique. Chaque participant a rempli le Self-Perceived Burden Scale (SPBS), l’Interpersonal Needs Questionnaire Perceived Burdensomeness Scale (INQPBS), l’Échelle d’idéation suicidaire de Beck et l’énoncé portant sur les pensées d’automutilation du Questionnaire sur la santé des patients-9.

**Résultats:** Tant les mesures du SPBS que celles de l’INQPBS étaient monofactorielles et présentaient une bonne cohérence interne. Elles présentaient une corrélation significative l’une avec l’autre (*r* = 0,50, p < 0,001) et avec les scores obtenus pour les deux mesures de l’idéation suicidaire (*r* = de 0,29 à 0,62, *p* < 0,001), Toutefois, l’INQPBS présentait une plus grande corrélation avec l’idéation suicidaire que le SPBS. Dans les analyses de régression, l’INQPS était un prédicteur de variance unique dans l’idéation suicidaire après rajustement en fonction du SPBS. Inversement, le SPBS ne contribuait pas de manière unique lorsque l’INQPBS était inscrit en premier.

**Conclusions**: Les patients souffrant de douleur chronique qui croient qu’ils sont devenus « un fardeau pour les autres » sont à plus grand risque d’idéation suicidaire. Les similarités et les différences conceptuelles entre les construits de perception de soi comme un fardeau et de fardeau perçu sont examinées afin d’expliquer pourquoi le fardeau perçu est le meilleur prédicteur de ce risque accru.

## Introduction

Two areas of research have shown that people who feel that they have become a “burden to others” are at increased risk for suicidal ideation (SI). The first area of research has focused on medical patients with physical disabilities, some of whom develop the belief that their functional limitations cause undue difficulty for caregivers and significant others. This belief has been termed *self-perceived burden*, and it has been defined as “a multidimensional construct arising from care recipients’ feelings of dependence and the resulting frustration and worry, which then lead to feelings of guilt at being responsible for the caregiver’s hardship” (p. 111).^1^ The prevalence of a problematic sense of self-perceived burden is quite high in some medical populations, ranging from 26% to 65% among patients receiving palliative care for cancer,^2–6^ 65% to 70% among stroke survivors,^7,8^ and over 70% among patients entering treatment for chronic nonmalignant pain.^9^ Self-perceived burden has been correlated with measures of pain, disability, and depression in different patient groups. In the palliative care setting, it has been associated with expressions of a desire for death and an interest in receiving physician-assisted suicide.^2,10,11^ In chronic pain, it has been associated specifically with self-reports of SI.^9,12,13^

The second body of research is grounded in the interpersonal theory of suicide,^14,15^ which proposes that serious SI depends on the emergence of two psychological precursor conditions, namely, *thwarted belongingness* and *perceived burdensomeness*. Thwarted belongingness refers to an unmet need for a sense of social connection and is reflected in issues such as isolation, loneliness, and impoverished social networks. Perceived burdensomeness refers to the belief that one has become such a problem for significant others that they would be better off if the individual was gone. Perceived burdensomeness “comprises two dimensions of interpersonal functioning—beliefs that the self is so flawed as to be a liability on others and affectively laden cognitions of self-hatred.” (p. 583)^15^ Perceived burdensomeness has been correlated with SI in studies of various groups,^16^ including college students,^17^ clients of counseling centers,^17^ military veterans,^18^ older adults,^19^ and patients with chronic pain.^20,21^

There are clear similarities between self-perceived burden and perceived burdensomeness, but there are also differences (see Table 1). Both constructs are thought to be multidimensional, and both emphasize the individual’s self-attribution of being the source of hardship for others. Self-perceived burden, however, relates specifically to individuals with illnesses or disabilities whose medical circumstances necessitate caregiving or other forms of instrumental support. Perceived burdensomeness is a broader concept and pertains to relationships more generally; one need not have a functionally limiting physical problem in order to experience perceived burdensomeness. Whereas self-perceived burden refers to concurrent feelings of frustration, worry, and guilt, perceived burdensomeness suggests a stronger affective component of self-hatred. Self-perceived burden makes no assumption of a specific cognitive inference that significant others would be better off if the individual was gone, whereas this inference is viewed as a critical pathway linking perceived burdensomeness to SI.

**Table 1.** Comparing and contrasting self-perceived burden and perceived burdensomeness.

Conceptual feature	Self-perceived burden	Perceived burdensomeness
Nature of construct	Multidimensional	Two-dimensional
Caregiver emphasis	Assesses patients’ perceptions of being aburden to caregivers	Assesses perception of being a burdengenerally in relationships
Interpersonal concern	Responsible for caregiver’s hardship	Liability on others
View of self	Dependent	Flawed
Affective response	Frustration, worry, guilt	Self-hatred
Others better off if dead/gone	Makes no reference	Important link to suicidal ideation

Self-perceived burden and perceived burdensomeness are also assessed with different measures. Self-perceived burden has been evaluated in medical patients using visual analog scales^22^ and semistructured interviews,^2,3,5,6,11^ but more commonly with the Self-Perceived Burden Scale (SPBS).^1^ Although the SPBS was developed as a 25-item questionnaire, this long version correlates highly with both ten-item^1^ and nine-item^23,24^ short forms that have been used in most research to date. Perceived burdensomeness, on the other hand, has usually been measured with the Interpersonal Needs Questionnaire (INQ).^17^ Although different forms of the INQ are available, a six-item version of the Perceived Burdensomeness Scale (INQPBS) has been recommended for research purposes.^25^

The aim of the present investigation was to examine the concordance between the constructs of self-perceived burden and perceived burdensomeness in a clinical sample of patients receiving treatment for chronic pain. Individually, these constructs arose independently of one another from research traditions in physical health and mental health settings. Conceptually they share much in common, but they have yet to be examined together in the same patient group. In this study, we examined the psychometric characteristics of the SPBS and INQPBS; the extent of their intercorrelation; their associations with measures of pain, mood, and function; and their differential prediction of SI. We proposed that self-perceived burden and perceived burdensomeness are, in fact, largely overlapping constructs. Given this overlap, we hypothesized that the SPBS and INQPBS would not both be significant concurrent predictors in a regression analysis of SI because they would account for the same variance.

## Materials and methods

### Participants and procedure

Participants were recruited from an interdisciplinary chronic pain management program at The Ottawa Hospital Rehabilitation Centre, a publicly funded tertiary care facility. Eligibility criteria for the program require a history (>3 months) of chronic pain that is not being considered for further interventional or surgical procedures. Referrals are generally made when medical treatment options have been exhausted but the patient remains limited in function and has difficulty coping.

Upon entry to the program, all patients complete a battery of questionnaires that are used for clinical monitoring, program evaluation, and research purposes. For the research component, each participant provided written informed consent for their questionnaires to be entered into a deidentified database. The protocol was approved by Ottawa Health Research Network Research Ethics Board.

Data from the participant group have been reported previously in a related project testing the interpersonal theory of suicide in patients with chronic pain.^21^ In that study, we reported that perceived burdensomeness, as measured by the INQPBS, was the strongest individual predictor of SI among a range of demographic (age, sex), pain-related (pain intensity, duration, distribution, functional limitations), cognitive (pain self-efficacy, pain catastrophizing), and psychological (depression, hopelessness, insomnia, thwarted belongingness) risk factors. The present study addresses the specific question of the relationship between self-perceived burden and perceived burdensomeness, which was not the focus of the previous research.

### Measures

#### Self-perceived burden

The SPBS^1^ measures the extent to which medical patients feel that their need for instrumental, emotional, or financial support places a strain on their caregivers or significant others. The SPBS was developed originally as a 25-item questionnaire and validated with patients undergoing dialysis for end-stage renal disease.^1^ A ten-item short form has been used in most research, although a nine-item alternative appears to have better psychometric properties.^23,24^ Respondents are asked to rate each item on a five-point scale, ranging from 1 (*none of the time*) to 5 (*all of the time*). In the current study, we administered the ten-item SPBS and found excellent internal consistency for both the ten-item (*α *= 0.93) and nine-item (α = 0.94) versions.

#### Perceived burdensomeness

Perceived burdensomeness was measured using the INQ, which assesses both perceived burdensomeness and thwarted belongingness, two core constructs of the interpersonal theory of suicide.^14,15^ The perceived burdensomeness factor includes six items. Participants are asked to answer each item on a seven-point scale, ranging from 1 (*not at all true for me*) to 7 (*very true for me*), with total scores ranging from 1 to 42. In the current sample, internal consistency for the INQPBS was high (*α* = 0.90).

#### Suicidal ideation

Each participant completed two measures of SI: the Beck Scale for Suicide Ideation (BSS) and Item 9 of the Patient Health Questionnaire-9 (PHQ-9). The BSS is a 21-item questionnaire.^26^ For each item, respondents are asked to provide a three-point symptom severity rating that ranges from 0 to 2. The BSS includes five initial screening questions, with items 4 and 5 assessing active and passive SI, respectively. If participants do not endorse any SI on either item 4 or 5, they are instructed to skip ahead to items 20 and 21, which inquire about past suicide attempts. Participants who do endorse SI on item 4 or 5 are instructed to complete the questionnaire in its entirety. Severity of SI is calculated by summing the first 19 items (excluding items 20 and 21). Total scores of the BSS range from 0 to 38, with higher scores indicating more severe SI. In previous research, the BSS has been found to have good internal consistency, as well as concurrent and predictive validity for future suicide attempts.^27^ In the current study, the internal consistency was excellent, *α *= 0.94.

SI was also assessed using Item 9 of the PHQ-9,^28^ which asks about the frequency of thoughts of death or self-harm. Participants respond on a four-point scale, ranging from 0 (*not at all*) to 3 (*nearly every day*).

#### Depression

Symptoms of depression were evaluated with the remaining eight items of the PHQ-9.^28^ Using a four-point scale, ranging from 0 (*not at all*) to 3 (*nearly every day*), participants are asked to indicate the frequency with which they experience the diagnostically relevant symptoms of major depression.^29^ A total score was calculated by summing the eight items, resulting in scores that range from 0 to 24. This eight-item scale correlates highly with the complete PHQ-9.^29^ In the current sample, internal consistency was good (*α* = 0.84).

#### Thwarted belongingness

Thwarted belongingness is a second key concept of the interpersonal theory of suicide. It was measured using the remaining items of the INQ (INQ Thwarted Belongingness Scale, INQTBS).^17,25^ The INQTBS contains nine items for this purpose, and participants respond to each using a seven-point rating scale anchored at 1 (*not at all true for me*) and 7 (*very true for me*). Total scores on the INQTBS range from 1 to 63.The internal consistency of the measure was excellent (*α* = 0.90).

#### Hopelessness

Hopelessness is a well-established risk factor for SI and suicidal behavior among psychiatric patients.^30,31^ In this study, hopelessness was assessed using the Hopelessness Assessment in Illness Questionnaire (HAIQ).^32^ Participants are asked to respond to eight items, each of which represents some aspect of the expression of a hopeless attitude. Responses are recorded on a three-point scale, ranging from 0 to 2, with higher scores indicating greater endorsement of hopelessness. Total scores range from 0 to 16. The HAIQ has shown good reliability and validity when compared to other measures of the hopelessness construct, but it may be more appropriate for use with medical patients with intractable problems.^32^ In the current study, internal consistency was high (*α* = 0.85).

#### Pain intensity

Pain intensity was assessed with four items that ask participants to rate their current pain, as well as their worst, least, and average pain over the past week. Each item is rated on an 11-point scale, ranging from 0 (*no pain*) to 10 (*as intense as you could imagine*). A total score was calculated by summing the responses. Past research has demonstrated good test–retest reliability for this measure, with composite scores providing better psychometric characteristics than individual items.^33^ In the current study, internal consistency was high (*α* = 0.89).

#### Pain catastrophizing

The 13-item Pain Catastrophizing Scale (PCS) was used to measure the degree to which individuals ruminate about pain, feel helpless in response to it, and magnify its consequences.^34^ For each item, individuals are asked to rate the frequency of occurrence of various pain-related thoughts and concerns. Responses to the five-point scale range from 0 (*not at all*) to 4 (*all of the time*). A total score was created by summing the responses. The PCS correlates with measures of pain intensity, mood, functional interference, and SI^13^ and has excellent test–retest reliability.^34^ In the current study, the PCS demonstrated excellent internal consistency (*α* = 0.93).

#### Pain self-efficacy

The Pain Self-Efficacy Questionnaire (PSEQ) was used to assess participants’ confidence in their ability to engage in and enjoy activities despite pain.^35^ Each of ten items is rated on a seven-point scale, ranging from 0 (*not at all confident*) to 6 (*completely confident*), and higher total scores reflect increasing self-efficacy. Previous investigations have found that the PSEQ correlates with measures of disability due to chronic pain and that it is associated with SI.^35,36^ In the current study, the internal consistency was high (*α* = 0.89).

#### Functional limitations

Functional limitations were assessed by a 16-item questionnaire that inquires about the degree of difficulty patients experienced when performing a variety of daily activities (e.g., dressing oneself, making meals) over the past two weeks. The questionnaire is a slightly modified version of one distributed by the International Association for the Study of Pain.^37^ Participants are asked to rate each item on a five-point scale, ranging from 1 (*no difficulty*) to 5 (*unable to do*). A total score was calculated, which could range from 1 to 80. This measure of functional limitation has been found to show good internal consistency and sensitivity to change.^9,38^ The internal consistency in this sample was excellent (*α* = 0.91).

#### Statistical Analyses

The statistical analyses comprised three parts. First, the psychometric characteristics of the SPBS and the INQPBS were examined, including rates of completion, item characteristics, score distributions, principal component structure, the conjoint correlation of the measures with one another, and their correlation with the measures of SI.

Second, the main hypothesis was tested in a series of hierarchical multiple regression analyses. Two analyses each were run using the BSS and PHQ-9 Item 9 as the criterion variables. In the first analysis, SPBS scores were entered on the initial step of the model. INQPBS scores were then entered on the second step in order to determine whether they could predict any unique residual variance. In the second analysis, the order of entry of the SPBS and the INQPBS scores was reversed.

The third aspect of the analyses examined correlations between the SPBS, the INQPBS, and other measures of pain, mood, and function. We compared the magnitudes of these correlations using Steiger’s *z* procedure, in order to determine whether self-perceived burden and perceived burdensomeness might correlate differentially with variables other than SI.

## Results

### Recruitment

As reported previously,^21^ 331 patients were enrolled in the chronic pain management program during the data collection period, 312 of whom consented to participate in the research (94.3%). To be included in the present analyses, participants had to have sufficient data on each of the SPBS, INQPBS, BSS, and Item 9 of the PHQ-9. We excluded 36 participants because of missing data for the SPBS, three because of the INQPBS, seven because of the BSS, and one because of missing Item 9 of the PHQ-9. A further five participants had missing data on more than one measure. In total, 52 individuals (16.6%) were excluded, resulting in a final sample of 260 participants. The rate of noncompletion was significantly higher for the SPBS than for the INQPBS (McNemar’s χ^2^ = 33.62, *P* < 0.001).

For the INQPBS and SPBS, a series of chi-square analyses and *t*-tests was conducted to determine whether participants who completed all questionnaires differed from those who did not on demographic and clinical characteristics. For the INQPBS, there were no differences between the two groups on any of these variables. For the SPBS, those who did not complete the measure were more likely to be single, χ^2^(5, *N* = 269) = 42.53, *P* < 0.001, older (52.43 years vs. 48.50 years), *t*(310) = 2.04, *P* < 0.05, and to have longer duration of pain (11.39 years vs. 8.24 years), *t*(303) = 2.06, *P* < 0.05.

### Sample characteristics

Of the 260 participants, 178 were women and 82 were men. They ranged in age from 19 to 84 years (M = 48.15, SD = 11.38) and the majority (85.6%) identified as Caucasian. Most participants were married or in a common-law relationship (70.8%). With respect to employment, 34.8% of participants were receiving either temporary or permanent disability benefits, 21.1% were working on a full- or part-time basis, 16.8% were unemployed, and 10.1% were retired. The majority had some postsecondary education (80.4%). The average duration of chronic pain was 8.17 years (SD = 8.19), and the primary pain sites included the back (47.3%), limbs (30.0%), neck (14.2%) or diffuse, widely distributed pain (4.2%).

### Psychometric properties of the SPBS and INQPBS

#### Self-Perceived Burden Scale

Scores for the ten-item SPBS ranged from 10 to 50, the lowest and highest possible values. The item with the highest mean score was “I feel guilty about the demands that I make on my caregiver,” whereas the item with the lowest mean score was “I am concerned that it costs my caregiver a lot of money to care for me.” For the total score, the mean was 27.55 (SD = 10.47), with 71.3% of the sample scoring 20 or higher. This cutoff score has been suggested as the most appropriate for identifying individuals who have a clinically elevated level of self-perceived burden.^3^

A principal components analysis of the ten items indicated a one-factor structure with an eigenvalue of 6.32, which explained 63.2% of the variance. However, item 7 of the SPBS, the only reverse-scored item (“I am confident that my caregiver can handle the demands of caring for me”), had relatively weak psychometric characteristics. Specifically, it showed a component loading of only 0.21 and an item-total correlation of *r* = 0.29. Simmons^24^ obtained similar results and recommended dropping this item in favor of a nine-item SPBS alternative. When we evaluated the revised version in a principal components analysis, the single-factor solution (eigenvalue = 6.28) explained 69.8% of the variance. The correlation between the nine- and ten-item versions was *r* = 0.99, indicating a high degree of convergence. Given the stronger psychometric properties of the nine-item scale, it was the version used in subsequent analyses.

The mean, standard deviation, and component loading for each individual item of the nine-item SPBS are shown in Table 2. The mean total score of the 9-item SPBS was 24.71 (*SD* = 10.19). A cut-off score of 18 showed excellent agreement with the cut-off score of 20 for the full 10-item scale, *κ* = .95.

**Table 2.** Properties of the Self-Perceived Burden Scale.^a^

Item	M (SD)	Component loading	Item-total correlation	Correlation with INQPBS^b^
(1) I worry that the health of my caregiver could suffer as a result of caring for me.	2.76 (1.37)	0.81	0.80	0.36
(2) I worry that my caregiver is overextending himself/herself in helping me.	2.76 (1.34)	0.87	0.86	0.36
(3) I am concerned that it costs my caregiver a lot of money to care for me.	2.18 (1.42)	0.61	0.64	0.35
(4) I feel guilty about the demands that I make on my caregiver.	3.19 (1.33)	0.89	0.88	0.40
(5) I am concerned that I am “too much trouble” to my caregiver.	2.87 (1.37)	0.88	0.88	0.49
(6) I am concerned that because of my illness, my caregiver is having to do too many things at once.	2.84 (1.39)	0.88	0.88	0.36
(8) I think that I make things hard on my caregiver.	2.87 (1.33)	0.85	0.85	0.46
(9) I feel that I am a burden to my caregiver.	2.95 (1.41)	0.85	0.85	0.52
(10) I am concerned that my caregiver is helping me beyond their capacity.	2.29 (1.31)	0.85	0.85	0.42

^a^Each item is rated on a scale ranging from 1 (*none of the time*) to 5 (*all of the time*). Item 7 has been omitted. ^b^All correlations are significant at *P* < .001.

INQPBS = Interpersonal Needs Questionnaire Perceived Burdensomeness Scale.

#### Perceived burdensomeness

Total scores for the INQPBS ranged from 6 to 42 (the full scale range), with a mean of 12.44 (*SD* = 7.84). The mean, standard deviation, and component loading for each item are shown in Table 3. The item with the highest mean was “I think I make things worse for the people in my life,” whereas the item with the lowest mean was “I think the people in my life wish they could be rid of me.” A principal components analysis of the six items yielded a one-factor solution (eigenvalue 4.22), which explained 70.4% of the total variance.

**Table 3.** Properties of the Interpersonal Needs Questionnaire Perceived Burdensomeness Scale.^a^

Item	M (SD)	Component loading	Item-total correlation	Correlation with SPBS^b^
(1) These days, people in my life would be better off if I were gone.	2.10 (1.60)	0.88	0.89	0.43
(2) These days, the people in my life would be happier without me.	1.81 (1.39)	0.87	0.85	0.39
(3) These days, I think I am a burden on society.	2.72 (2.02)	0.82	0.85	0.45
(4) These days, I think my death would be a relief to the people in my life.	1.59 (1.27)	0.84	0.82	0.32
(5) These days, I think the people in my life wish they could be rid of me.	1.49 (1.16)	0.82	0.79	0.34
(6) These days, I think I make things worse for the people in my life.	2.78 (1.91)	0.79	0.83	0.49

^a^Each item is rated as a scale ranging from 1 (*not at all true for me*) to 7 (*very true for me*).

^b^All correlations are statistically significant at *P* < .001.

SPBS = Self-Perceived Burden Scale.

### Relationships between self-perceived burden and perceived burdensomeness

The SPBS and INQPBS total scores correlated moderately with one another, *r*(260) = 0.50, *P* < 0.001. Table 2 shows the correlations of the individual SPBS items with the total SPBS score, as well as the item correlations with the INQPBS total scores. Table 3 presents the comparable information for the INQPBS.

For the SPBS, item-total correlations ranged from *r *= 0.64 to *r* = 0.88. Individual SPBS items correlated with the INQPBS total score at levels ranging from *r* = 0.35 to *r* = 0.52.

Individual items on the INQPBS all correlated with the scale’s total score at levels exceeding *r* = 0.80, except for item 5. The correlations between these items with the SPBS total scores ranged from 0.32 to 0.49.

### Correlation between measures of SI

The two measures of SI, the BSS and Item 9 of the PHQ-9, were highly correlated with each other, *r*(260) = .77, *p* < .001. The *r*^2^ value indicates 59% shared variance, which suggests that they are assessing a common underlying construct but that they are not redundant.

### Self-perceived burden, perceived burdensomeness, and SI

Scores on the BSS were correlated significantly with those on both the SPBS, *r*(260) = 0.29, *P* < 0.001, and the INQPBS, *r*(260) = 0.55, *P* < 0.001; however, the correlation with the INQPBS was significantly higher, Steiger’s *z*(260) = −4.76, *P* < 0.001. SPBS and INQPBS scores were also correlated significantly with Item 9 of the PHQ-9, *r*(260) = 0.35, *P* < 0.001, and *r*(260) = 0.62, *P* < 0.001, respectively. Again, the correlation between Item 9 of the PHQ-9 and the INQPBS was significantly higher than that with the SPBS, Steiger’s *z*(260) = −5.28, *P* < 0.001.

### Regression analyses predicting SI

#### BSS

Two separate hierarchical multiple regression analyses were conducted to examine the relative contributions of the SPBS and INQPBS in predicting scores on the BSS. In the first analysis, the SPBS was entered in the initial step of the model. The model was significant, accounting for approximately 8% of the variance (*R*^2^ = 0.08, *F*_1, 258_ = 23.74, *P* < 0.001). Next, INQPBS scores were included, which led to a significant increase in the amount of explained variance (*R*^2^_change_ = 0.22, *F*_change 1, 257_ = 78.91, *P* < 0.001).

In the second analysis, the order of entry was reversed, with the INQPBS being entered before the SPBS. In step 1, INQPBS scores accounted for 29.9% of the variance (*R*^2^ = 0.29, *F*_1, 258_ = 109.99, *P* < 0.001). The SPBS was added in the second step but did not increase the amount of variance explained (*R*^2^_change_ = 0.00, *F*_change 1, 257_ = 0.18, *P* = 0.67).

#### PHQ-9 Item 9

The same regression analyses were then rerun using the PHQ-9 Item 9 as the criterion measure of SI. In the first analysis, the SPBS was entered in the first step of the model and accounted for approximately 12% of the variance in PHQ-9 Item 9 scores (*R*^2^ = 0.12, *F*_1, 258_ = 36.41, *P* < 0.00). The INQPBS was included next, which led to a significant increase in the amount of explained variance (*R*^2^_change_ = 0.26, *F*_change 1, 257_ = 109.73, *P* < 0.001).

In the second analysis, INQPBS scores were entered on the first step and accounted for 38.3% of the variance (*R*^2^ = 0.38, *F*_1, 258_ = 160.29, *P* < 0.001). In the second step, the SPBS was included, but it did not enhance the regression model significantly (*R*^2^_change_ = 0.00, *F*_change 1, 257_ = 1.12, *P* = 0.29).

### Correlations with measures of pain, mood, and function

As shown in Table 4, scores on both the SPBS and INQPBS were correlated significantly with measures of depression, thwarted belongingness, hopelessness, pain intensity, catastrophizing, self-efficacy, and functional limitations. When comparing differences between the SPBS and INQPBS in the magnitudes of these correlations, we used a Bonferroni-adjusted significance level of *P* < 0.007. In general, there were different patterns of correlation observed across variables. In the case of hopelessness, scores on the INQPBS were more strongly correlated than were those of the SPBS. For depression, thwarted belongingness, pain intensity, catastrophizing, and self-efficacy, the magnitudes of the correlations involving the SPBS vs. INQPBS were not significantly different from one another. For functional limitations, the SPBS was more strongly correlated than the INQPBS.

**Table 4.** Correlations between the Self-Perceived Burden Scale, the Interpersonal Needs Questionnaire Perceived Burdensomeness Scale, and measures of pain, mood, and function.

Measure	M (SD)	SPBS^a^	INQPBS^a^	Steiger’s *z* (260)	*P* value
PHQ-8	13.17 (5.75)	0.52	0.50	0.39	n.s.
INQTBS	30.25 (12.88)	0.43	0.53	−1.91	n.s.
HAIQ	6.89 (3.27)	0.47	0.62	−3.06	0.002
Pain intensity	24.01 (6.18)	0.20	0.23	−0.50	n.s.
PCS	24.98 (12.04)	0.36	0.45	−1.62	n.s.
PSEQ	25.38 (11.90)	−0.44	−0.42	−0.37	n.s.
Functional limitations	39.52 (9.57)	0.46	0.30	2.85	0.004

^a^All correlations are significant at *P* ≤ .001.

SPBS = Self-Perceived Burden Scale; INQPBS = Interpersonal Needs Questionnaire Perceived Burdensomeness Scale; PHQ-8 = Patient Health Questionnaire excluding item 9; INQTBS = Interpersonal Needs Questionnaire Thwarted Belongingness Scale; HAIQ = Hopelessness Assessment in Illness Questionnaire; PCS = Pain Catastrophizing Scale; PSEQ = Pain Self-Efficacy Scale. n.s. = nonsignificant.

## Discussion

The constructs of self-perceived burden and perceived burdensomeness both emphasize an individual’s self-attribution of having become a source of hardship for significant others. Beyond that, however, the constructs differ with regard to the circumstances in which they apply (physical disability vs. more broadly), the affective component of the experience (frustration and guilt vs. self-hatred), and the ultimate inference that other people would be better off if the individual was dead or gone. Both constructs have been related to SI, but they have never been examined concurrently in the same group of patients. Hence, it has remained unclear as to whether their associations with SI are overlapping, redundant, or unique.

The results of the principal components analysis indicate that both the SPBS and the INQPBS are best regarded as unidimensional scales, albeit with multiple facets. This finding is relevant because both self-perceived burden and perceived burdensomeness make reference to multidimensionality in their definitions. In the case of self-perceived burden, the dimensions refer to the experience of receiving care in the distinct domains of physical, emotional, and financial support. Indeed, Cousineau et al. initially designed the SPBS to be a multidimensional measure, but psychometric evaluation supported a more general burden factor.^1^ In the case of perceived burdensomeness, the definition identifies two dimensions: the recognition of a flawed self that is a liability on others and an accompanying sense of self-hatred. Although these dimensions are conceptually separable, in practice they comprise a single factor in the present version of the INQPBS.

We found that the SPBS had a higher noncompletion rate than the INQPBS. This appeared related to the specific requirements of the SPBS, which assesses respondents’ reactions to receiving care from others. Some individuals who lived alone indicated that they had no significant others who provided any form of instrumental support. Other respondents had broader social networks but did not consider the concepts of caregiving or care receiving to be relevant to the nature of their relationships. In these cases, the respondents could acknowledge being limited functionally by chronic pain but they did not regard themselves as requiring actual care. Hence, they viewed the SPBS as inapplicable to them. The instructions for the INQPBS, on the other hand, are broader and refer to individuals’ current beliefs and experiences with regard to themselves and other people; these beliefs and experiences are not limited to caregiving relationships.

Although self-perceived burden and perceived burdensomeness are related constructs, they are also distinct. For example, the two measures correlated moderately with one another, sharing about 25% of variance. Furthermore, with the exception of the discrepant item 7 of the SPBS, every item within each measure correlated more highly with its own scale total than with the total score of the other scale. In fact, the correlations with score totals were nonoverlapping; that is, the item-total correlations for the SPBS items were in the range of *r *= 0.62 to 0.88, which were higher in every case than the range of *r* = 0.35 to 0.52 exhibited by the items of the INQPBS.

As in previous research, both measures were correlated significantly with SI. The INQPBS had the highest correlation in this regard, a finding that was consistent across both the BSS and the PHQ-9 measures of SI. In the regression analyses, INQPBS scores accounted for unique variance in SI in all models, whereas SPBS scores did not. This served to disconfirm our initial hypothesis that self-perceived burden and perceived burdensomeness would be largely overlapping constructs and account for the same variance. Although the constructs do overlap in some ways, perceived burdensomeness apparently has certain characteristics that make it the more salient predictor of SI.

An examination of Table 1 offers some indication as to what these characteristics might be. In general, perceived burdensomeness refers to a stronger affective state than self-perceived burden. With self-perceived burden, an individual may recognize a degree of dependence on others and feel frustration, worry, and guilt about any hardship that may ensue as a result. However, this does not necessarily lead to a generalized sense of the self as flawed or to a state of self-hatred. These strong cognitive and affective responses do contribute to perceived burdensomeness, and they may be components that are especially predictive of SI. Moreover, with perceived burdensomeness, the individual may draw the further conclusion that others would be better off if that person was dead or gone. Again, the belief that suicide could produce an altruistic benefit to loved ones may be a powerful, direct link to SI, but it is not addressed specifically in the conceptualization or measurement of self-perceived burden.

From this conceptual comparison, one could conclude that perceived burdensomeness is a more important construct than self-perceived burden for health research in general. We caution, however, that the present study focused specifically on the issue of SI. Based on the correlations of the SPBS with the BSS and PHQ-9 measures of SI, we estimate that about 8% to 12% of the variance in SI is explained by self-perceived burden, almost all of which is subsumed by the 30% to 38% of variance explained by perceived burdensomeness. Nevertheless, there are many important health decisions that have little, if anything, to do with suicide but may be influenced by one’s concern for the welfare of others. For example, in a review of studies on self-perceived burden among patients with advanced illness, McPherson et al. noted an influence on such health decisions as foregoing life-sustaining treatment, preparing advance directives, and choosing hospital vs. home care.^10^ Interestingly, however, they also noted a strong association between self-perceived burden and requests for physician-assisted suicide among patients who were terminally ill.

The different patterns of correlation among the SPBS, INQPBS, and other pain-related variables are instructive in this regard. In addition to the measures of SI itself, scores on the INQPBS were more highly correlated with hopelessness, a well-established risk factor for SI. Other correlations, however, including those between the INQPBS, SPBS, and measures of depression, pain intensity, and pain-related cognitions were about equal in magnitude. Finally, in the case of functional limitations, the pattern was actually reversed, and the correlation with the SPBS was significantly higher than that of the INQPBS. Hence, whether self-perceived burden or perceived burdensomeness is most relevant in a given context may depend on the specific behavior or health decision under consideration. In the case of SI, perceived burdensomeness appears to be the more informative and predictive construct, but this might not hold for other clinical concerns.

It should be noted, however, that this suggestion is speculative and remains to be addressed in future research. The present study was focused on SI, so the findings are limited with respect to other aspects of health behavior. Moreover, the study group was composed mostly of individuals who were entering interdisciplinary rehabilitation treatment for chronic musculoskeletal pain. Further research will be required to determine whether the results can be generalized to groups with other types of pain or disability.

Clinically, this study clarifies the circumstances whereby some patients with chronic pain are at increased risk for SI. Chronic pain can have far-reaching functional, economic, and personal ramifications that contribute to one's belief of having become the cause of hardship for others. Although this belief is not universal among people with chronic pain, it is relatively common. When it is accompanied by the further inference that significant others would actually be better off if the individual was dead or gone, then clinicians should be especially vigilant to the emergence of SI. This recommendation is in keeping with predictions of the interpersonal theory of suicide.

## References

[1] Cousineau N, McDowell I, Hotz S, Hébert P. Measuring chronic patients’ feelings of being a burden to their caregivers. Med Care. 2003;41(1):110–118. doi:10.1097/01.MLR.0000039832.32412.7D. 12544548

[2] De Faye BJ, Wilson KG, Chater S, Viola RA, Hall P. Stress and coping with advanced cancer. Palliat Support Care. 2006;4(3):239–249. doi:10.1017/S1478951506060317. 17066965

[3] McPherson CJ, Wilson KG, Lobchuk MM, Brajtman S. Self-perceived burden to others: patient and family caregiver correlates. J Palliat Care. 2007;23(3):135–142. 18069434

[4] Tang ST, Hsieh CH, Chiang MC, Chen JS, Chang WC, Chou WC, Hou MM. Impact of high self-perceived burden to others with preferences for end-of-life care and its determinants for terminally ill cancer patients: a prospective cohort study. Psychooncology. 2016;26(1):102–108. doi:10.1002/pon.4107. 26950036

[5] Wilson KG, Chochinov HM, McPherson CJ, Skirko MG, Allard P, Chary S, Gagnon PR, Macmillan K, De Luca M, O’Shea F, et al. Desire for euthanasia or physician-assisted suicide in palliative cancer care. Health Psychol. 2007;26(3):314–323. doi:10.1037/0278-6133.26.3.314. 17500618

[6] Wilson KG, Curran D, McPherson CJ. A burden to others: a common source of distress for the terminally ill. Cogn Behav Ther. 2005;34(2):115–123. doi:10.1080/16506070510008461. 15986788

[7] McPherson CJ, Wilson KG, Chyurlia L, Leclerc C. The balance of give and take in caregiver–partner relationships: an examination of self-perceived burden, relationship equity, and quality of life from the perspective of care recipients following stroke. Rehabil Psychol. 2010;55(2):194–203. doi:10.1037/a0019359. 20496974

[8] Ren H, Liu C, Li J, Yang R, Ma F, Zhang M, Wang R, Gan L. Self-perceived burden in the young and middle-aged inpatients with stroke: a cross-sectional survey. Rehabil Nurs. 2014;41(2):101–111. doi:10.1002/rnj.193. 25471627

[9] Kowal J, Wilson KG, McWilliams LA, Péloquin K, Duong D. Self-perceived burden in chronic pain: relevance, prevalence, and predictors. Pain. 2012;153(8):1735–1741. doi:10.1016/j.pain.2012.05.009. 22703692 PMC3999031

[10] McPherson CJ, Wilson KG, Murray MA. Feeling like a burden to others: a systematic review focusing on the end of life. Palliat Med. 2007;21(2):115–128. doi:10.1177/0269216307076345. 17344260

[11] Wilson KG, Dalgleish TL, Chochinov HM, Chary S, Gagnon PR, Macmillan K, De Luca M, O’Shea F, Kuhl D, Fainsinger RL. Mental disorders and the desire for death in patients receiving palliative care for cancer. BMJ Support Palliat Care. 2016;6(2):170–177. doi:10.1136/bmjspcare-2013-000604. 24644212

[12] Fishbain DA, Bruns D, Bruns A, Gao J, Lewis JE, Meyer LJ, Disorbio JM. The perception of being a burden in acute and chronic pain patients is associated with affirmation of different types of suicidality. Pain Med. 2016;17(3):530–538. 26332796 10.1111/pme.12889

[13] Wilson KG, Kowal J, Henderson PR, McWilliams LA, Péloquin K. Chronic pain and the interpersonal theory of suicide. Rehabil Psychol. 2013;58(1):111–115. doi:10.1037/a0031390. 23438008 PMC3998981

[14] Joiner TE Jr. Why people die by suicide. Cambridge (MA): Harvard University Press; 2005.

[15] Van Orden KA, Witte TK, Cukrowicz KC, Braithwaite S, Selby EA. Joiner TE Jr. The interpersonal theory of suicide. Psychol Rev. 2010;117(2):575–600. doi:10.1037/a0018697. 20438238 PMC3130348

[16] Hill RM, Pettit JW. Perceived burdensomeness and suicide-related behaviors in clinical samples: current evidence and future directions. J Clin Psychol. 2014;70(7):631–643. doi:10.1002/jclp.22071. 24421035

[17] Van Orden KA, Cukrowicz KC, Witte TK, Joiner TE Jr. Thwarted belongingness and perceived burdensomeness: construct validity and psychometric properties of the Interpersonal Needs Questionnaire. Psychol Assess. 2012;24(1):197–215. doi:10.1037/a0025358. 21928908 PMC3377972

[18] Bryan CJ, Cukrowicz KC, West CL, Morrow CE. Combat experience and the acquired capability for suicide. J Clin Psychol. 2010;66(10):1044–1056. doi:10.1002/jclp.20703. 20821797

[19] Cukrowicz KC, Cheavens JS, Van Orden KA, Ragain RM, Cook RL. Perceived burdensomeness and suicide ideation in older adults. Psychol Aging. 2011;26(2):331–338. doi:10.1037/a0021836. 21401264 PMC3699192

[20] Kanzler KE, Bryan CJ, McGeary DD, Morrow CE. Suicidal ideation and perceived burdensomeness in patients with chronic pain. Pain Pract. 2012;12:602–609. doi:10.1111/j.1533-2500.2012.00542.x. 22429694

[21] Wilson KG, Heenan A, Kowal J, Henderson PR, McWilliams LA, Castillo D. Testing the interpersonal theory of suicide in chronic pain. Clin J Pain. 2017;33(8):699–706. doi:10.1097/AJP.0000000000000451. 27768608

[22] Chochinov HM, Kristjanson LJ, Hack TF, McClement S, Harlos M. Burden to others and the terminally ill. J Pain Symptom Manage. 2007;34(5):463–471. doi:10.1016/j.jpainsymman.2006.12.012. 17616329

[23] Oeki M, Mogami T, Hagino H. Self-perceived burden in patients with cancer: scale development and descriptive study. Eur J Oncol Nurs. 2012;16(2):145–152. doi:10.1016/j.ejon.2011.04.010. 21616716

[24] Simmons LA. Self-perceived burden in cancer patients. Cancer Nurs. 2007;30(5):405–411. doi:10.1097/01.NCC.0000290816.37442.af. 17876187

[25] Hill RM, Rey Y, Marin CE, Sharp C, Green KL, Pettit JW. Evaluating the Interpersonal Needs Questionnaire: comparison of the reliability, factor structure, and predictive validity across five versions. Suicide Life Threat Behav. 2015;45(3):302–314. doi:10.1111/sltb.12129. 25308815

[26] Beck AT, Steer RA. Manual for the Beck Scale for Suicide Ideation. San Antonio (TX): Psychological Corporation; 1991.

[27] Beck AT, Brown GK, Steer RA. Psychometric characteristics of the Scale for Suicide Ideation with psychiatric outpatients. Behav Res Ther. 1997;35(11):1039–1046. doi:10.1016/S0005-7967(97)00073-9. 9431735

[28] Kroenke K, Spitzer RL, Williams JB, Löwe B. The Patient Health Questionnaire Somatic, Anxiety, and Depressive Symptom scales: a systematic review. Gen Hosp Psychiatry. 2010;32(4):345–359. doi:10.1016/j.genhosppsych.2010.03.006. 20633738

[29] American Psychiatric Association. Diagnostic and statistical manual of mental disorders. 4th ed. Washington (DC): American Psychiatric Association; 2000.

[30] Beck A, Steer R, Kovacs M, Garrison B. Hopelessness and eventual suicide: a 10-year prospective study of patients hospitalized with suicidal ideation. Am J Psychiatry. 1985;142(5):559–563. doi:10.1176/ajp.142.5.559. 3985195

[31] Glanz LM, Haas GL, Sweeney JA. Assessment of hopelessness in suicidal patients. Clin Psychol Rev. 1995;15(1):49–64. doi:10.1016/0272-7358(94)00040-9.

[32] Rosenfeld B, Pessin H, Lewis C, Abbey J, Olden M, Sachs E, Amakawa L, Kolva E, Brescia R, Breitbart W. Assessing hopelessness in terminally ill cancer patients: development of the Hopelessness Assessment in Illness Questionnaire. Psychol Assess. 2011;23(2):325–336. doi:10.1037/a0021767. 21443366 PMC3717574

[33] Jensen MP, Turner JA, Romano JM, Fisher LD. Comparative reliability and validity of chronic pain intensity measures. Pain. 1999;83(2):157–162. doi:10.1016/S0304-3959(99)00101-3. 10534586

[34] Sullivan MJL, Bishop SR, Pivik J. The Pain Catastrophizing Scale: development and validation. Psychol Assess. 1995;7(4):524–532. doi:10.1037/1040-3590.7.4.524.

[35] Nicholas MK. The Pain Self-Efficacy Questionnaire: taking pain into account. Eur J Pain. 2007;11(2):153–163. doi:10.1016/j.ejpain.2005.12.008. 16446108

[36] Nicholas MK, McGuire BE, Asghari A. A 2-item short form of the Pain Self-Efficacy Questionnaire: development and psychometric evaluation of PSEQ-2. J Pain. 2015;16(2):153–163. doi:10.1016/j.jpain.2014.11.002. 25463701

[37] International Association for the Study of Pain Task Force on Records and Data Retrieval. Pain Database Questionnaire. Seattle (WA): International Association for the Study of Pain; 1995.

[38] Kowal J, Wilson KG, Geck CM, Henderson PR, D’Eon JL. Changes in perceived pain severity during interdisciplinary treatment for chronic pain. Pain Res Manage. 2011;16(6):451–456. doi:10.1155/2011/817816. PMC3298050 22184556

